# The in vitro *Mycobacterium bovis* BCG Moreau infection of human monocytes that induces Caspase-1 expression, release and dependent cell death is mostly reliant upon cell integrity

**DOI:** 10.1186/s12950-019-0223-1

**Published:** 2019-07-15

**Authors:** Paulo R. Z. Antas, Carlos G. G. Ponte, Matheus R. Almeida, Lawrence H. P. Albuquerque, Periela S. Sousa-Vasconcelos, Thaíze Pedro, Natália L. S. Gomes, Otacílio C. Moreira, Fernanda C. Silva, Luíz R. R. Castello-Branco, Rosa T. Pinho

**Affiliations:** 1 Laboratório de Imunologia Clínica, Oswaldo Cruz Institute, Fiocruz, Pavilhao Leonidas Deane, 4th Floor, Av. Brasil 4365, Rio de Janeiro, RJ 21045-900 Brazil; 2 Laboratório de Biologia Molecular e Doenças Endêmicas, Oswaldo Cruz Institute, Fiocruz, Rio de Janeiro, Brazil; 3 Gaffree Guinle State University Hospital of Rio de Janeiro, Rio de Janeiro, Brazil

**Keywords:** BCG vaccine, Tuberculosis, Monocyte Apoptosis, qPCR

## Abstract

**Background:**

Caused by *Mycobacterium tuberculosis*, tuberculosis (TB) is an extremely contagious disease predominantly affecting the lungs. TB is found worldwide and has a major impact on public health safety primarily due to its high mortality rate. Applied for over a hundred years as a preventive measure, *Mycobacterium bovis* BCG remains the only available TB vaccine. Only one seminal study about the apoptotic pathways induced by this vaccine in the monocytic lineage of the host cell has found the effects of BCG on regulation of apoptosis. The aim of this study was to explore beyond that pioneer study the pathway related to the in vitro cell-death pattern and the inflammatory response to the BCG vaccine in human monocytes.

**Methods:**

Cohorts of HIV-negative volunteers were enrolled: adult Healthy Donors (HD) and neonates’ Umbilical Cord Blood (UCB) individuals. Host mononuclear cells were infected with the *M. bovis* Moreau strain of BCG vaccine at 16, 24, 48, and 72 h. The Real-Time RT-PCR for TRADD, Bcl-2, and Caspases-1 and -3 were performed, and supernatants were assayed in parallel for Caspase-1, NLRP3, HO-1, and IL-1β levels whereas caspases were assessed intracellularly. The effect of a BCG infection in monocytes was characterized via a metabolic activity assay by LDH release profiles.

**Results:**

Overall, the BCG vaccine induced significantly higher Caspase-1 and Bcl-2 mRNA levels in both the HD and UCB groups (*p*-value ≤0.05). In addition, a significant increase solely in Caspase-1 protein levels was also noted in both HD and UCB (*p*-value ≤0.05) notwithstanding the absence of any damaged cell membranes.

**Conclusions:**

Our data directly corroborate other findings showing that BCG Moreau led to an increased secretion of IL-1β but not IL-18, two Caspase-1-activated cytokines, and are also in support of the model that the BCG Moreau infection of human mononuclear cells may induce a cell-death pattern involving Caspase-1 activation.

**Electronic supplementary material:**

The online version of this article (10.1186/s12950-019-0223-1) contains supplementary material, which is available to authorized users.

## Background

The World Health Organization (WHO) estimated there were 10.4 million new tuberculosis (TB) cases along with 1.4 million related deaths in 2015 [1]. Although the number of obits worldwide from TB - a disease caused by *Mycobacterium tuberculosis* - actually decreased by 22% between 2000 and 2015, it continues to be the biggest infectious human killer and one of the top ten causes of deaths globally. For the most part, TB is most prominent in the developing world, as in the 22 highest-burden countries in Asia, Africa, and South America, responsible for a full 82% of all estimated TB cases in the world [1]. This alarming result is directly associated with poor treatment outcomes and the development of a series of multidrug-resistant strains, not to mention the deadly combination of *M. tuberculosis* and the human immunodeficiency virus (HIV) co-infection [1]. However, while there has been much progress in both basic and clinical research over the past decade, the lack of progress in understanding host immunity to adult pulmonary TB is increasingly recognized as a major impediment to both the control and eventual elimination of the disease [2]. Additional preventive efforts, including studies involving the effectiveness of the BCG vaccine against TB, are urgently required.

Even though vaccination with the attenuated live *M. bovis* bacille Calmette-Guérin (BCG) verifiably induces protective immune responses in children against the most severe and fatal forms of TB, it is ineffective against pulmonary TB in hyper-endemic countries and has had a negative impact on the global TB crisis [3]. To this day, BCG is the only licensed TB vaccine available, having been administered globally on a yearly basis since 1921 to an estimated 100 million children. In addition, new TB vaccines have been developed over time but in the absence of any real understanding regarding the protective immune mechanisms.

The current prophylactic vaccine strategy relies on replicating BCG bacilli in an effort to trigger a long-lasting immune response. The maintenance of specific immunity essentially needs BCG bacilli to produce antigens primarily associated with cell-wall compounds [4]. The BCG strain used in Brazil is the one referred to as Moreau. But, again, very little is known about its long-lasting protective properties [5].

The BCG vaccine is not a single organism, but consists of substrains that vary in genotypes and phenotypes. Since the 1940s and because the serial passage of this vaccine in different countries after initial seed distribution from the Pasteur Institute in France, substantial sequence modifications in terms of specific insertions and deletions in the genome among the BCG substrains have been described and speculated to result in differences in immunological activities [6]. Of note, a sequential emergence of the parental BCG vaccine substrains revealed a total of 24 BCG substrains currently in circulation worldwide. Then, systematizing the BCG vaccine strains may facilitate our understanding of protection provided by BCG immunizations. Among others, this topic has been fully revisited and commented by an expert in the field [7]. There is a lack of substantiated in vitro studies attempting to elucidate the protective mechanisms afforded by the BCG Moreau strain. As the TB epidemic continues to rage, more attention has been given to the direct applicability and improvement of existing vaccination and managerial strategies and the development of new ones.

Inflammasomes are intracellular multiprotein complexes that, in response to danger signals, trigger the biological maturation of pro-inflammatory cytokines [8]. Thus, to induce high IL-18 and IL-1β productions in monocytes, the canonical pathway of the NLRPs/ASC-Caspase-1 axis is also required. NLRP3 inflammasome activation promptly accelerates Caspase-1 maturation in conjunction with the resulting functional IL-18 and IL-1β. However, a recent study has challenged whether Caspase-1 activation necessarily occurs primarily as an intracellular event, which is thus highly dependent on cell integrity [9].

Contrariwise, heme oxygenase-1 (HO-1) plays an opposing, anti-inflammatory role [8]. As a hemoglobin component, free heme is only released when either hemolysis or extensive cell damage such as necrosis occurs, resulting in an inflammatory response. To lessen the ensuing damage, HO-1 is induced as a ubiquitous, cytoprotective enzyme, whose products exhibit certain protective biological activities including anti-inflammatory and antioxidant activities to counter oxidative cellular stress [8].

In 1997, a seminal study firstly established the effects of mycobacteria on regulation of apoptosis in mononuclear cells [10]. In view of the fact that since then limited data are currently available and because the macrophagic/monocytic lineages in the lungs represent the first line of defense against incoming airborne pathogens in the developing granuloma, the present study focused on more thoroughly exploring the pathways related to the in vitro cell-death pattern associated with the inflammatory response to the BCG Moreau strain in human monocytes.

The major original hypothesis was that BCG-infected host cells upregulate apoptotic pathway candidates at a molecular level. The additional hypothesis was that activation of Caspase-1 downstream is fully dependent on cellular integrity, occurring at an extracellular level in BCG-infected human mononuclear cell cultures. Both the inflammatory (NLRP3) and anti-inflammatory compounds (HO-1) potentially released during BCG-host cell interactions were also compared. The contention was that a better understanding of the changes induced by the BCG infection could help identify the resulting protective processes and thereby open up future prospects for improved vaccines. Furthermore, it was hoped that the present work would result in an enhanced overall understanding of the pathogenesis of tuberculosis.

## Patients and methods

### Study population

In the present study, the specimens tested were collected between November 2010 and July 2016 from two groups of donors enrolled at different sites in the City of Rio de Janeiro. The first group of specimens (buffy coats) was from adult healthy donors (HD: totaling 43 individuals) found in the blood bank of the Clementino Fraga Filho Federal University Hospital (anonymous donations from individuals aged ≥18 years). The second group of specimens was from newborns’ umbilical cord blood samples (UCB: a total of 25 neonates), collected by way of umbilical cord puncture procedures on full-term placentas from disease-free mothers who had had uncomplicated deliveries at the Gaffree Guinle State University Hospital (HUGG). The UCB cells were collected promptly afterward. The HUGG-UNIRIO (protocol # 59117015.0.0000.5258) and the IOC-Fiocruz (protocol # 35775014.0.0000.5248) institutional review boards approved the study procedures. All study participants (healthy adults, mothers of neonates, public health authorities, and the children’s parents or guardians) voluntarily provided their written informed consent.

### Mononuclear cell purification and co-culture

Both Peripheral Blood (PBMC) and Cord Blood Mononuclear Cells (CBMC) were separated within no more than 24 h (average 5 h) after obtaining and then culturing blood specimens from all study participants, as described elsewhere [11]. In vitro infections (approximately 1 × 10^7^ viable bacilli per vial) with the Moreau BCG vaccine (Ataulpho de Paiva Foundation-FAP, Rio de Janeiro) of freshly-isolated PBMCs and CBMCs (5 × 10^6^ cells each) were performed for 16 h (exclusively for gene expression) and then for 24, 48, and 72 h, at a multiplicity of infection (MOI) of 2:1 (bacilli:host cell). Prior assessment had established that the infection rates remained comparable for MOI 1:1, 2:1 and 10:1. Cultured mononuclear cells in a RPMI medium (Sigma Immunochemicals, USA) supplemented with 10% human AB serum were kept at 37 °C in a humidified 5% CO_2_ atmosphere in individual 12 × 75 mm sterile polystyrene tubes (Falcon, Corning Inc., USA). Previous experiments with these tubes showed better cell viability when compared to conventional culture plates [12]. Both the PBMCs and CBMCs used for subsequent gene expression analysis were submitted to *Trizol*-based RNA purification (Life Technologies, Grand Island, NY, USA) followed by RNA extraction using the RNeasy Mini kit (Qiagen, Austin, TX, USA) and subsequently transcribed via the Superscript VILO kit (Invitrogen, Carlsbad, CA, USA). The culture supernatants were stored at − 70 °C for further use in the protein detection assays. A parallel process according to Lima et al. (2015) [13] was performed to establish the acute monocytic human leukemia cell line THP-1 in a monocytic-like state. Cells were co-cultured as described above and assayed for gene expression and Caspase-1 extracellular detection according to the protocol detailed below.

### Gene expression by qRT-PCR

Real-time quantitative PCR (qRT-PCR) assays were performed in a ABI Prism 7500 Fast Sequence Detection System, using single-tube TaqMan gene expression assays (Applied Biosystems, USA) for the human targets, as follows: Caspase-1, an apoptotic effector pathway (Hs 00354836-m1); Caspase-3, another apoptotic effector pathway (Hs 00234387-m1); Bcl-2, an anti-apoptotic molecule (Hs 00608023-m1); TRADD, an adapter protein (Hs 00182558-m1) together with the endogenous housekeeping control genes glyceraldehyde 3-phosphate dehydrogenase (GAPDH, Hs 99999905-m1) and 18S rDNA (Hs 99999901-s1). Assays were performed according to the manufacturer’s instructions. The cycling conditions for qRT-PCR were: 95 °C for 10 min, followed by 45 cycles at 95 °C for 15 s and 60 °C for 1 min. Reactions were performed in duplicate using 2 μL of a cDNA template for a total volume of 20 μL. The relative quantification of gene expression was performed using the ΔΔCt method [14]. PCR assays were performed in triplicate.

### Polycaspase and Caspase-1 detection by FACS

FAM-FLICA commercial kits (FAM-VAD-FMK-polycaspase and FAM-YVAD-FMK-Caspase-1, Immunochemistry Technologies, Bloomington, MN, USA) were first individually dissolved in 50 μL of dimethyl sulfoxide (DMSO) and then diluted by adding 200 μL of sterile phosphate buffered saline (PBS), pH 7.4. Finally, fluorescent signals derived from the intracellular-bound FLICA inhibitor probes were run separately and analyzed in a flow cytometer device (FACScalibur, BD, USA).

### Detection of Caspase-1, NLRP3, HO-1 and IL-1β using ELISA

The Caspase-1 (Quantikine ELISA Human Caspase-1/ICE, R&D Systems, USA), the nucleotide-binding domain and leucine-rich repeat protein-3 (NLRP3) inflammasome (Human NACHT, LRR and PYD Domains- containing Protein 3 NLRP3/C1orf7/CIAS1/NALP3/PYPAF1, R&D Systems, USA), the enzyme heme oxygenase (HO)-1 (DuoSet Human Total HO-1/HMOX1, MyBiosource Inc., USA), and the IL-1β (DuoSet Human IL-1 beta/IL-1F2, R&D Systems, USA) activities in cell-free culture supernatants were assayed by human enzyme-linked immunosorbent assay (ELISA) commercial kits, as specified by the respective manufacturers.

### LDH detection

The CytoTox-ONE Homogeneous Membrane Integrity kit (Promega, Southampton, UK), a non-radioactive cytotoxicity assay, was used to measure the amount of lactate dehydrogenase (LDH) induced by the BCG vaccine and released in the mononuclear cell cultures. Cell-free supernatants were thawed out once, assessed under each of four conditions (baseline; pooled BCG-infected cells; a positive control by means of Lysis Solution; and BCG-infected cells plus a positive control) and subsequently assayed on a black 96-well plate after adding 50 μL of CytoTox-ONE Reagent (1:1 ratio). Samples were then incubated in the dark for 10 min at room temperature before 25 μL of stop solution was added. Fluorescence was measured immediately at a 525 nm excitation wavelength and 580–680 nm emission wavelengths using a microplate reader.

### Statistical evaluation

Data were compared within the groups by the Mann-Whitney U test using PRISM version 5.01 for Windows (GraphPad Software, USA). The *p*-value was scored and considered significant when ≤0.05. Data were reported in medians and interquartile ranges (IQR). Spearman’s rank correlation coefficient was calculated to analyze the relationship of Caspase-1 and IL-1β levels in response to the BCG vaccine.

## Results

### Gene expression detection

**Fig. 1:**
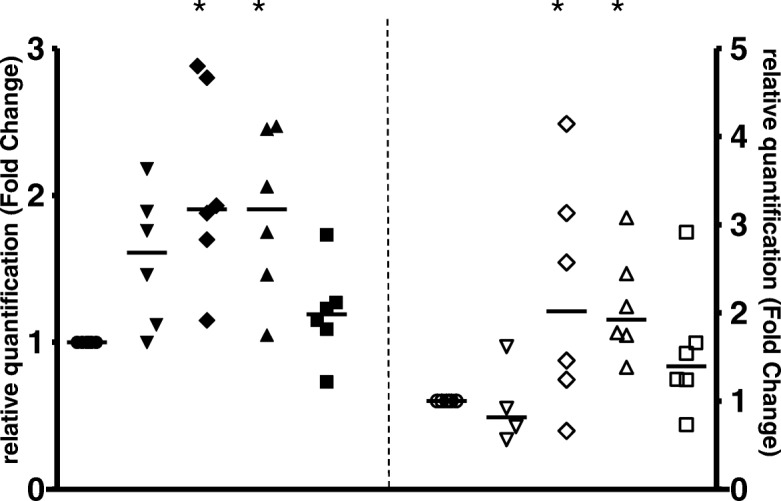
The expressions of Baseline (circle), TRADD (lower triangle), Bcl-2 (diamond), Caspases-1 (upper triangle), and − 3 (square) mRNA levels in healthy donor (closed symbols) and umbilical cord blood (opened symbols) groups after 16 h of in vitro Moreau BCG vaccine infection of human mononuclear cells. Data points denote individual donors and horizontal bars represent median values in each condition. *p-value ≤0.05, according to the Mann-Whitney U test

In TB, the specific cell-death pattern plays a critical role in the ensuing immune response [15]. Previously, we have found that healthy donor adults (HD) vaccinated with BCG-Moreau during childhood (BCG vaccination in Brazil is mandatory after birth) display a distinct in vitro cell-death pattern when compared with umbilical vein cells from naïve individuals (UCB) who had never been exposed to the mycobacterium before [11, 13, 16]. In order to investigate these data at the molecular level, cells were 1) infected with the BCG-Moreau vaccine for 16 h (yielding 87% live bacilli on average after reconstitution) or 2) cells were left resting (baseline) uninfected. Then, an assessment was made of the mRNA levels of Caspases-1 and -3, TRADD and Bcl-2. As such, a significant increase (*p*-value ≤0.05) in Caspase-1 and Bcl-2 mRNA in both the HD and UCB groups was detected (Fig. 1). Conversely, and coincidently, there was no substantial difference in the amplifications of Caspase-3 or TRADD mRNA in either the HD nor UCB groups. As expected, the THP-1 cell line run in parallel reflected HD and UCB groups (i.e., the 8.3; 1.4; 7.1 and 4.8 mRNA levels of TRADD, Bcl-2, and Caspases-1 and -3, respectively).

### Polycaspase and Caspase-1 detection

To provide support for the molecular mechanism found during the in vitro BCG-infected mononuclear cell gene expression experiments, detection of broad, functional, intracellular caspase activity in a real-time fashion was prioritized. To do so, fresh cells were assayed using a commercially available approach based on a polycaspase inhibitor conjugated to carboxyfluorescein. It had been suggested that this compound could possibly be successful in the live detection of apoptotic activity [17]. However, no significant difference in the polycaspase levels found after 24 and 48 h of BCG-infected cultures vs. those under baseline conditions was identified in the HD group (Table 1). Polycaspase inhibitor activity was not re-tested in the UCB group because of cost constraints. Likewise, we were unable to perform further microscopy images intent to resolve a representative qualitative data for the degree of staining for polycaspase and Caspase-1.

**Table 1:** Polycaspase and Caspase-1 intracellular staining (%) in mononuclear cells of healthy donor adults (HD, *n* = 7) and newborns’ umbilical cord bloods (UCB, *n* = 8) groups

	Polycaspase	Caspase-1	
	HD	HD	UCB
Negative Control	63.1 & 20.9^a^	36.8 & 63.3	4.0 & 11.3
BCG 24 h	60.9 & 33.1	59.2 & 23.0	7.4 & 23.5
BCG 48 h	70.7 & 10.2	46.2 & 80.8	9.7 & 31.7
H/C Shock Control	nd	59.1 & 18.0	6.0 & 88.9

^a^Median & IQR

**Fig. 2:**
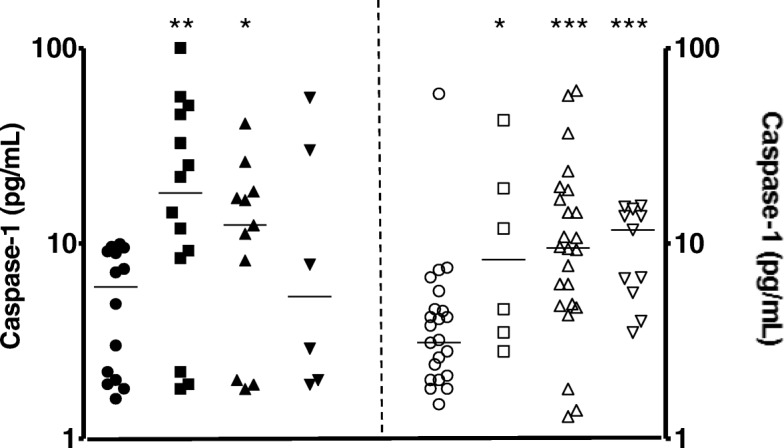
Caspase-1 levels (pg/mL) in cell-free supernatants of healthy donor (Y1, closed symbols) and umbilical cord blood (Y2, opened symbols) groups representing the baseline, uninfected cells (circle), and the Moreau BCG vaccine in vitro infection of human mononuclear cells in 24 (square), 48 (upper triangle), and 72 h (lower triangle). Data points denote individual donors and horizontal bars represent median values in each condition. *p-value ≤0.05; **p-value ≤0.01; ***p-value ≤0.001, according to the Mann-Whitney U test

Motivated by the high mRNA levels of Caspase-1 in both groups in the preceding qRT-PCR experiments, the Caspase-1 released in the BCG Moreau-infected cultures of the HD and UCB groups at 24, 48, and 72 h using ELISA [note: similar time points were assessed for the subsequent biomarkers as well] was evaluated. In fact, it is now clear that, in live-cell models, Caspase-1 activation occurs while undergoing cellular release and is, therefore, decidedly not an intracellular event (which is in agreement with our prior polycaspase data) but a typical free and mediated Caspase-1 activation in response to an exogenous inflammasome inducer [9]. In line with the above, after BCG infection, there were significant differences (*p*-value ≤0.05) between the higher Caspase-1 protein levels in the HD and UCB groups (Fig. 2). As expected, the THP-1 cell line was reproduced in both groups studied (13.1% & 3.2 vs. 3.0% & 2.3, at 48 h of BCG Moreau-infected THP-1 cell-line vs. baseline conditions, respectively: *p*-value = 0.02). Accordingly, the data mirrored not only the earlier qRT-PCR results (Fig. 1), but also those regarding the IL-1β levels (*p*-value ≤0.01) in both groups (Additional file 1: Figure S1).

**Fig. 3:**
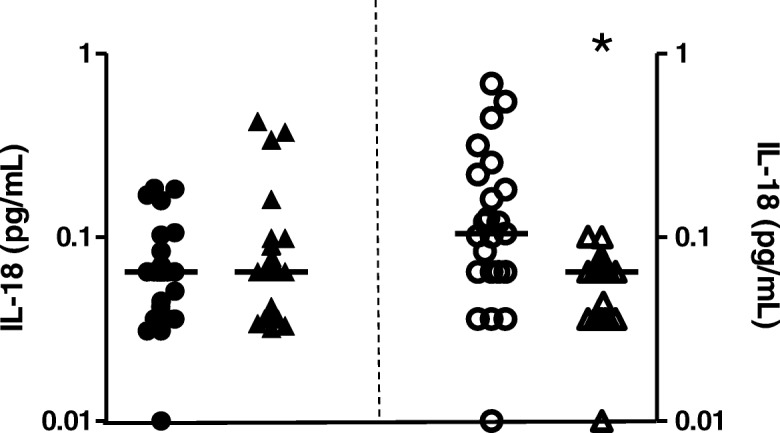
IL-18 levels (pg/mL) in cell-free supernatants of healthy donor (Y1, closed symbols) and umbilical cord blood (Y2, opened symbols) groups representing the baseline, uninfected cells (circle), and Moreau BCG vaccine in vitro infection of human mononuclear cells in 48 h (upper triangle). Data points denote individual donors and horizontal bars represent median values in each condition. *p-value ≤0.05, according to the Dunn’s paired test [11]

It has been shown earlier that the chain of events leading to stimulation of a cytokine storm by mycobacteria is dependent of the proinflammatory milieu involving IL-18 secreted from monocytes and, like IL-1β, induced by a Caspase-1-dependent pathway [18]. On the other hand, our previous study of circulating biomarkers found that active IL-18 plasma levels positively correlated with inflammatory IL-23 in cord blood, but not in adult blood [19]. Thus, IL-18 released during 48 h-BCG infection of the HD and UCB groups were previously assayed [11], and the results were found to be differentially induced (Fig. 3).

Owing to the previous supernatant data, an in vitro functional Caspase-1 assay was concurrently performed in order to detect its intracellular activity in a real-time fashion in both PBMC and CBMC. Again, fresh cells were re-assayed using a specific Caspase-1 inhibitor conjugated to carboxyfluorescein. Likewise, no detectably significant change in Caspase-1 protein levels after either 24 or 48 h of BCG-infected cultures (or even when Heat and Cold shocks [H-C protocol in [11]] were induced) vs. baseline conditions was found in the mononuclear cells of either group (Table 1).

### Detection of lytic cells

**Fig. 4:**
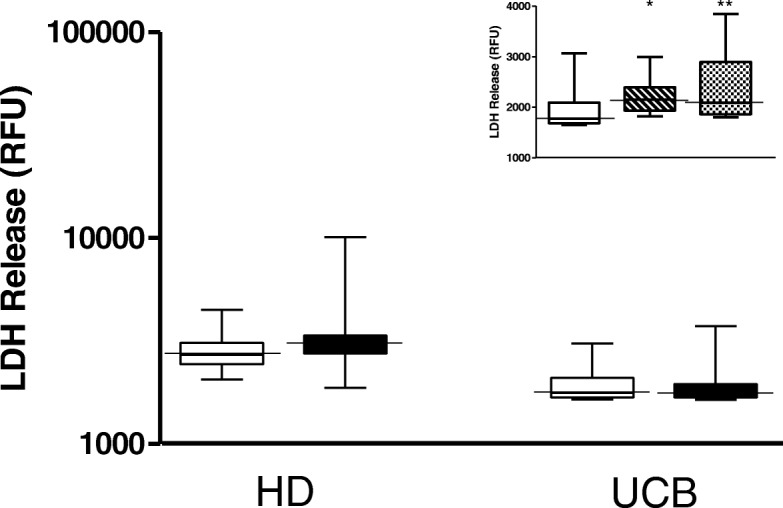
The box and whisker plots for LDH release in cell-free supernatants of healthy donor (*n* = 9) and umbilical cord blood (*n* = 13) groups induced in vitro by Moreau BCG vaccine infection of human mononuclear cells. The open boxes depict the baseline uninfected cells (Ctl). The insert illustrates the positive control (hatched boxes) and BCG-infected cells plus the positive control (dotted boxes). Boxes represent the median, 25th to 75th percentile ranges while whiskers show maximum and minimum values. *p-value ≤0.05 vs. Ctl; **p-value ≤0.01 vs. Ctl

To estimate the number of necrotic cells, it has become widely accepted that the measurement of leakage constituents from the cytoplasm into the surrounding culture medium is an effective approach [20]. It was thus decided to directly evaluate the necrotic levels induced by the BCG vaccine in mononuclear cells. Research over the last decade has indicated that caspases also play previously unexpected roles in regulating necrotic cell death [21]. So, other than the observations that BCG induced Caspase-1 enhancement at both the molecular and protein levels in supernatants, but not intracellularly, no significant difference was observed in either group with regard to LDH release when comparing BCG-infected cells with those in the baseline condition. On the other hand, the positive control run in parallel worked suitably (Fig. 4). Hence, due to lack of leakage, it is assumed that the cultures in the present study maintained their normal cell integrity.

### Inflammatory pathway detection

It was first contended that the in vitro BCG infection of human mononuclear cells might result in high NLRP3 inflammasome activation, an event opposed to anti-inflammatory HO-1 induction [8]. In fact, seminal studies had identified NLRP3 as belonging to a large family of innate, internal, intracellular immune receptors only able to recognize PAMPs in cytoplasmic compartments (Reviewed by [22]). However, as previously stated, these compounds must be released in cell-free supernatants that are reliant on the necrotic level to be measurable. As such, the NLRP3 and HO-1 levels induced by the BCG-Moreau vaccine were not higher in either group. Actually, a discrete but significant decrease (*p*-value ≤0.05) in BCG-infected neonate cell NLRP3 vs. baseline levels was noticed at a later time point (Additional file 2: Figure S2). These resulting data are in line with a prior finding that no LDH was released after induction by the BCG vaccine (Fig. 4).

## Discussion

It has been shown that an avirulent mycobacterium is able to induce macrophagic apoptosis and that the inhibition of this critical host mechanism might be seen as an evasive strategy by the pathogen (Reviewed by [23]). Conversely, necrosis, induced by virulent *M. tuberculosis* to skew the protective host immune response, could be considered an effective strategy in this regard. Alternatively, pyroptosis, a form of programmed pro-inflammatory cell death similar to necrosis, has been recognized as Caspase-1 dependent [24]. In fact, Caspase-1 mRNA transcripts showed a substantial increase in fresh, human BCG-infected monocytes as well as immortalized THP-1 monocytes in vitro in both study groups. These increases were followed by the rise of Caspase-1, found again in the HD and UCB cultures. It may be contemplated that this datum is a sort of proof-of-concept.

Nevertheless, our data related to the absence of intracellular caspase staining is intriguing since others have already assessed cytoplasmic polycaspases in a similar system [25]. There are major debatable technical issues with regard to the above-mentioned and present studies. These discrepancies, however, most likely result from the length of time the cell cultures were evaluated, i.e., at 5 vs. 24 h (the earliest), respectively. As a whole, the present Caspase-1 data corroborate both current and previous findings, which have shown persistently significant production of BCG vaccine-induced, mature IL-1β [11, 16]. Thus, the high IL-1β levels assessed in a variety of studies during the in vitro BCG infection of monocytes, particularly among HD individuals, were consistent with the increased rate of apoptosis, but not of necrosis. However, it should be borne in mind that, relative to fresh human blood monocytes, human macrophages are deficient in their ability to process and release IL-1β, a critically M-CSF-dependent mechanism [26]. In this regard, both quantitative and qualitative differences between monocytes from newborns and adults were duly revisited in our previous publication [16]. Besides, the present report also corroborated the in vitro modulation of the inflammatory response through the distinct release of the bioactive IL-18, another Caspase-1-activated cytokine, in adults and neonates upon monocyte infection using the BCG vaccine [11]. Likewise, differential ex vivo IL-18 levels between these cohorts have been formerly reported in the plasma [19].

To date, pyroptosis seems to play a significant role in specific biological systems. Yet, the advantages of pyroptotic-generated cell deaths are still controversial. While there is evidence that pyroptosis may benefit the host in a given infection, it can be harmful during sepsis [27]. Thus, Caspase-1 activation cleared intracellular *Legionella pneumophila* and *Burkholderia thailandensis* in vivo in an IL-1β-independent manner and can be deemed to be an efficient bactericidal mechanism of the innate immune system [28]. The Gasdermin D membrane pore formation constitutes the effector mechanism of pyroptotic cell death [29]. Although past and current evidence is profuse, additional studies still warrant in-depth inspection into whether Gasdermin D plays any critical role in our system.

In the present study, a significant increase in the Bcl-2 mRNA transcript was encountered in both the HD and UCB groups at 16 h following infection with BCG vaccine. This finding is in contrast with the result obtained in another in vitro model of mycobacterial infection of mononuclear cells, in which Bcl-2 mRNA was downregulated in monocytes in shorter time points (between 2 and 6 h of BCG infection), and Bcl-xL, another inhibitor of apoptosis, was minimally upregulated by this vaccine [10]. In that study, the authors concluded that apoptosis may be mediated by downregulation of Bcl-2. Studies involving this molecule and the BCG vaccine are quite scarce. As previously stated, it has been shown that pathogenic *M. tuberculosis* infection inhibits the intrinsic apoptotic pathway in the host cell and is believed to involve such anti-apoptotic molecules as Bcl-2 [30]. One potential assumption may be that, while there is high Bcl-2 expression (at least at the transcriptional level), a counter effect of pro-apoptotic molecules like Bad, which binds to Bcl-2, may upregulate apoptosis in the end [31].

In a recent study, bacterial lipopolysaccharide induced the overexpression of NLRP3 and, consequently, of Caspase-1 as well [8]. However, when HO-1 was induced, the formation of the NLRP3 inflammasome was blocked. Thus, the induction of HO-1 attenuated the inflammatory damage in human cells in vitro through the inhibition of inflammasome activation. Another contemporary study found that IL-1β secretion was heme-induced by an activating NLRP3 inflammasome in macrophages [32]. Most importantly, the expressive increase in heme levels and activation of the NLRPs/ASC-Caspase-1 axis were detected after the murine ureteral blockade was inhibited by forced HO-1 expression. Thus, heme also seems to be a potentially dangerous activator of the NLRP3 inflammasome, which actually plays a key role in IL-1β secretion during experimental kidney inflammation. It is noteworthy that, at least in the present study, no consistent levels of either inflammatory (NLRP3) or anti-inflammatory (HO-1) candidates in the cell-free host supernatants upon BCG infection have been found. It is also conceivable to associate the previous finding with the failure of the BCG vaccine to induce the release of LDH from the host-cell cytosol while the overlying plasma membrane seems to remain undamaged. Of interest, a previous study done with kidney tubule has shown that glycine limits LDH release after addition of alpha-toxin to form a plasma membrane cytolytic oncotic pore in already anoxic proximal tubules (Reviewed by [33]). Current datasheet of our RPMI medium support this model, since about 10 mg/mL of glycine has been added to supplement this culture medium. Taken together, these assessments well illustrate some opposing features of inflammatory vs. anti-inflammatory compounds, which, in conjunction with a LDH bioassay in a controlled glycine buffer solution, surely requires further investigation.

We admit that a major limitation of the present study was the failure to include additional targets, i.e., other than the four evaluated here and related to the in vitro cell-death pathway for assessment at the gene expression level. It is, therefore, reasonable to speculate that although we have found BCG-induced, anti-apoptotic Bcl-2 mRNA upregulation, pro-apoptotic members of the same family of regulator proteins, namely, Mcl-1, Bak, BCL2L11/Bim, PMAIP1/NOXA, and BBC3/PUMA, might seize Bcl-2 later and downregulate its expression [31]. If correct, this critical step of blocking the protective effects of the programmed cell-death inhibitor Bcl-2 may ultimately drive the BCG-infected mononuclear cell apoptosis into the HD group. Lastly, in our view, another limitation of this study was neglecting to ascertain the actual cell-death mechanism induced by the BCG vaccine taking place in neonates.

## Conclusion

In this study, the Moreau strain of the BCG vaccine induced the highest Caspase-1 and Bcl-2 expression at the transcriptional level in both the HD and UCB groups. Likewise, there was a significant increase in Caspase-1 release at the protein level. A marked dependency on cell integrity to convert Caspase-1 extracellularly was also noted in vitro in both individuals. Our data directly corroborate those in which BCG Moreau induced increased IL-1β secretion, one of the Caspase-1-activated cytokines, since the correlation analysis between those two biomarkers was found to be strongly positive as well as showing direct BCG-induced modulation. The study also recapitulated a differential in vitro inflammatory response through the release of IL-18 levels induced by the BCG vaccine in the HD and UCB groups. However, additional studies are warranted to better scrutinize firstly, the lack of relationship between IL-18 and IL-1β levels in the two cohorts studied, and secondly, the actual cell-death mechanism as to whether necrosis or pryoptosis is taking place in neonates [11, 16]. These findings support the hypothesis that BCG Moreau infection of both mycobacterial-sensitized and -naïve human mononuclear cells is responsible for inducing a cell-death pattern involving Caspase-1 activation.

## Additional files

Additional file 1: **Figure S1.** (A) IL-1β levels (pg/mL) in cell-free supernatants of healthy donor (Y1, closed symbols) and umbilical cord blood (Y2, opened symbols) groups representing the baseline, uninfected cells (circle), and Moreau BCG vaccine in vitro infection of human mononuclear cells in 48 h (upper triangle). (B) The relationship between Caspase-1 and IL-1β levels during the in vitro inflammatory responses to the BCG Moreau vaccine in the healthy donor group, analyzed by linear regression at 48 h, showed a positive association. Data points denote individual donors and horizontal bars represent median values in each condition. **p-value ≤0.01; ***p-value ≤0.001, according to the Mann-Whitney U test, and ^#^*p*-value = 0.03, according to Spearman’s rank correlation coefficient. (PPT 167 kb)

Additional file 2: **Figure S2.** (A) NLRP3 and (B) HO-1 levels (pg/mL) in cell-free supernatants of healthy donor (Y1, closed symbols) and umbilical cord blood (Y2, opened symbols) groups representing the baseline, uninfected cells (circle), and the Moreau BCG vaccine in vitro infection of human mononuclear cells in 24 (square), 48 (upper triangle), and 72 h (lower triangle, except for B). Data points denote individual donors and horizontal bars represent median values in each condition. *p-value ≤0.05, according to the Mann-Whitney U test. (PPT 161 kb)

## Data Availability

The data used to support the findings of this study are available from the corresponding author upon request.

## Abbreviations

BCG: Bacillus Calmette-Guerin
CBMC: Cord blood mononuclear cell
DMSO: Dimethyl sulfoxide
ELISA: Enzyme linked immunosorbent assay
FACS: Fluorescence-activated cell sorting
GAPDH: Glyceraldehyde 3-phosphate dehydrogenase
HD: Healthy donors
HIV: Human immunodeficiency virus
HO-1: Heme oxygenase-1
IL-18: Interleukin-18
IL-1β: Interleukin-1 beta
IQR: Interquartile ranges
LDH: Lactate dehydrogenase
MOI: Multiplicity of infection
NLRP3: Nucleotide-binding domain and leucine-rich repeat protein-3
PBMC: Peripheral blood mononuclear cell
qRT-PCR: quantitative Real Time-PCR
TB: Tuberculosis
UCB: Umbilical Cord Blood
WHO: World Health Organization
