# Supplementary figures and images for: The in vitro Mycobacterium bovis BCG Moreau infection of human monocytes that induces Caspase-1 expression, release and dependent cell death is mostly reliant upon cell integrity
Source: J Inflamm (Lond). 2019 Jul 15;16:18. doi: 10.1186/s12950-019-0223-1 (PMC6633651; doi:10.1186/s12950-019-0223-1)

## Slide 1
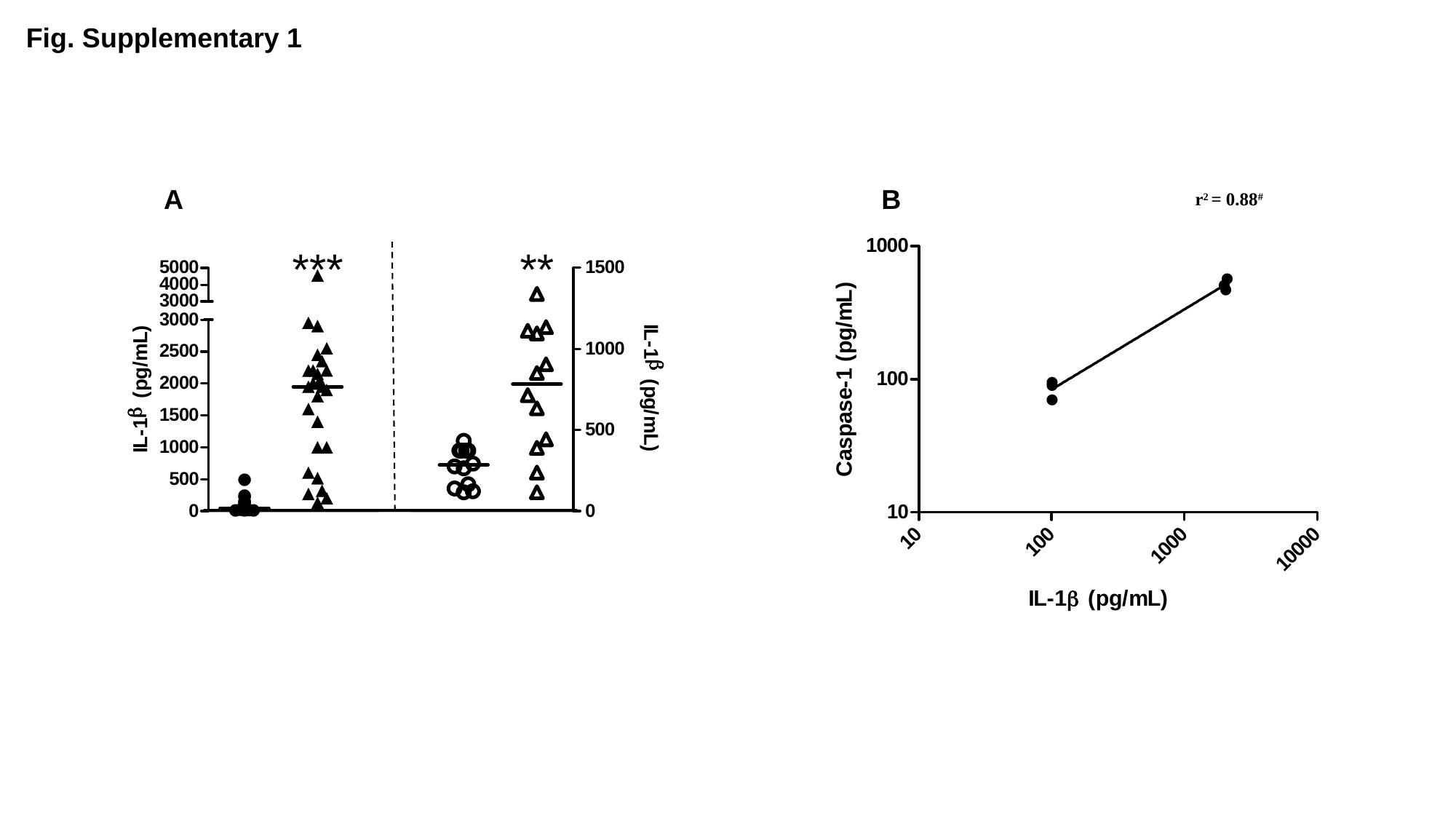

Fig. Supplementary 1
A
B
r2 = 0.88#

Supplement: Supplementary file 1 — Figure S1. (A) IL-1β levels (pg/mL) in cell-free supernatants of healthy donor (Y1, closed symbols) and umbilical cord blood (Y2, opened symbols) groups representing the baseline, uninfected cells (circle), and Moreau BCG vaccine in vitro infection of human mononuclear cells in 48 h (upper triangle). (B) The relationship between Caspase-1 and IL-1β levels during the in vitro inflammatory responses to the BCG Moreau vaccine in the healthy donor group, analyzed by linear regression at 48 h, showed a positive association. Data points denote individual donors and horizontal bars represent median values in each condition. **p-value ≤0.01; ***p-value ≤0.001, according to the Mann-Whitney U test, and #p-value = 0.03, according to Spearman’s rank correlation coefficient. (PPT 167 kb) [file 12950_2019_223_MOESM1_ESM.ppt]

## Slide 1
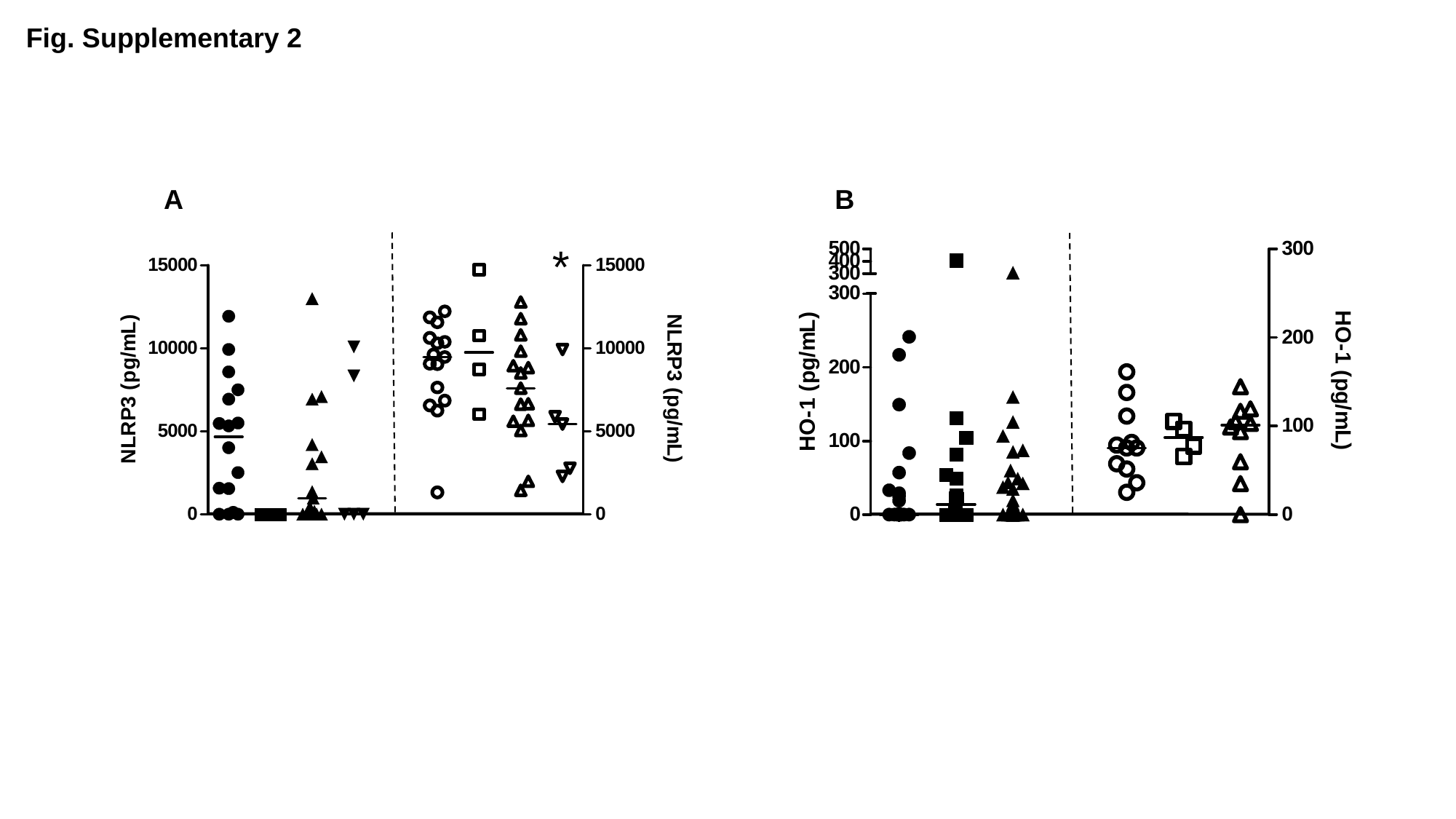

Fig. Supplementary 2
A
B

Supplement: Supplementary file 2 — Figure S2. (A) NLRP3 and (B) HO-1 levels (pg/mL) in cell-free supernatants of healthy donor (Y1, closed symbols) and umbilical cord blood (Y2, opened symbols) groups representing the baseline, uninfected cells (circle), and the Moreau BCG vaccine in vitro infection of human mononuclear cells in 24 (square), 48 (upper triangle), and 72 h (lower triangle, except for B). Data points denote individual donors and horizontal bars represent median values in each condition. *p-value ≤0.05, according to the Mann-Whitney U test. (PPT 161 kb) [file 12950_2019_223_MOESM2_ESM.ppt]
